# A Novel Recurrent Neural Network-Based Ultra-Fast, Robust, and Scalable Solver for Inverting a “Time-Varying Matrix”

**DOI:** 10.3390/s19184002

**Published:** 2019-09-16

**Authors:** Vahid Tavakkoli, Jean Chamberlain Chedjou, Kyandoghere Kyamakya

**Affiliations:** Institute for Smart Systems Technologies, University Klagenfurt, A9020 Klagenfurt, Austria; jean.chedjou@aau.at (J.C.C.); kyandoghere.kyamakya@aau.at (K.K.)

**Keywords:** matrix inversion, time-varying matrix, noise problem in time-varying matrix inversion, recurrent neural network (RNN), RNN-based solver, real-time fast computing

## Abstract

The concept presented in this paper is based on previous dynamical methods to realize a time-varying matrix inversion. It is essentially a set of coupled ordinary differential equations (ODEs) which does indeed constitute a recurrent neural network (RNN) model. The coupled ODEs constitute a universal modeling framework for realizing a matrix inversion provided the matrix is invertible. The proposed model does converge to the inverted matrix if the matrix is invertible, otherwise it converges to an approximated inverse. Although various methods exist to solve a matrix inversion in various areas of science and engineering, most of them do assume that either the time-varying matrix inversion is free of noise or they involve a denoising module before starting the matrix inversion computation. However, in the practice, the noise presence issue is a very serious problem. Also, the denoising process is computationally expensive and can lead to a violation of the real-time property of the system. Hence, the search for a new ‘matrix inversion’ solving method inherently integrating noise-cancelling is highly demanded. In this paper, a new combined/extended method for time-varying matrix inversion is proposed and investigated. The proposed method is extending both the gradient neural network (GNN) and the Zhang neural network (ZNN) concepts. Our new model has proven that it has exponential stability according to Lyapunov theory. Furthermore, when compared to the other previous related methods (namely GNN, ZNN, Chen neural network, and integration-enhanced Zhang neural network or IEZNN) it has a much better theoretical convergence speed. To finish, all named models (the new one versus the old ones) are compared through practical examples and both their respective convergence and error rates are measured. It is shown/observed that the novel/proposed method has a better practical convergence rate when compared to the other models. Regarding the amount of noise, it is proven that there is a very good approximation of the matrix inverse even in the presence of noise.

## 1. Introduction

Matrix inversion is extensively used in linear algebra (e.g., for solving linear equations). Although matrix inversion is already referred to in very ancient books, tremendous attention has been devoted to it (by scientists) mainly since the 17th century. The interest devoted to matrix inversion has led to the development of various methods, concepts, and algorithms for solving linear equations [1]. Solving matrix inversion is very useful in engineering, physics, and other natural sciences [2]. Solving a real-time/online matrix inversion is part of mathematics and control theory. It finds important applications in various areas such as traffic simulation and/or online control in the frame of intelligent transportation systems, robotics (e.g., for kinematics and inverse kinematics), communications [3], machine learning [4], smart/complex antennas (MIMO) [5,6,7], Field Programmable Gate Array (FPGA) [8,9], signal processing [10], image processing [11,12], and robotics [13,14,15], etc.

Also, the matrix inversion is very useful in several decision-making algorithms. There are plenty of different heuristics and goal-programming algorithms which are using linear relationships to solve problems and they often try to solve those problems via matrix inversion; this is the case, for example, in social media networks where it can help to speed up the ranking amongst different categories [16,17].

Matrix inversion is further widely used and is of critical importance in various processing contexts in smart sensors. In some cases, the accuracy of certain measurements can be significantly improved by involving matrix inversion. For example, a real-time matrix inversion is used to ensure high-precision in multi parameter sensing while using the so-called fiber Bragg grating (FBG)-based sensors [18]. Thereby, in that related approach, the sensor functionality is approximated with a linear system and the problem is thus solved through linear system of equations. Also, this last-mentioned approximation has other advantages like the capability of re-constructing lost information, a capability used, for example, in the context of so-called compressed sensing [19,20].

In general, the measurements of different physical quantities related to a dynamical system can be explained as linear measurement equations or multi-variate sensing processes [21]. The measurement of those/such mentioned quantities often requires a real-time computing of matrix inversions. Further, the relationship between different measurements can also be used for calibrating purposes for the related sensors [22].

Finally, matrix inversion used in sensors related processing processes does also provide the capability to significantly reduce noise in measurements. For example, just for illustration, matrix inversion is helping to ignore noisy measurement in spatially distributed sensors [23].

To fulfil the expected role in various applications, different methods have been developed to achieve both fast convergence and higher accuracy of the matrix inversion related calculations. Some of the most famous methods are the following: elimination of variables, Gaussian elimination (also known as row reduction), lower-upper (LU) decomposition, Newton’s method, eigenvalue decomposition, Cholesky decomposition, and many other methods.

Generally, one can categorize matrix inversion methods into two different groups: (a) the recursive (or iterative) methods; and (b) the direct methods [7,24,25,26].

The first group encompasses methods like Gauss–Seidel or gradient descent. The initial condition (or starting point) is provided and each step uses the last value to calculate the new value. In each iteration, the solution approximation becomes better until the desired accuracy is reached [27,28].

On the other hand, direct methods like Cholesky or Gaussian elimination typically compute the solution in finite number of iterations. They can find the exact solution if there exists no rounding error [29]. Those analytical methods normally have a minimum arithmetic complexity of $O\left(n^{3}\right)$ (provided those algorithms are implemented on a single CPU). For parallel systems, this arithmetic complexity is different, as the algorithm should be changed in a way to fit for efficient processing on a parallel system architecture. Hereby, depending on the parallelizability potential of the algorithm, the present number of parallel cores will affect the final effective complexity. However, in real benchmarking implementations, most parallel implementations of algorithms do require the transfer of large amounts of data amongst processors and thereby this communication need does lead to a loss of efficiency of the algorithm with respect to speeding-up capability in presence of multiple cores. Therefore, implementing the same algorithm on a parallel system is very inefficient (i.e., speed-up). On the other hand, in practice, the noise problem is a very serious problem and the related denoising process is an expensive one, which can provoke a violation of the systems’ real-time property. The need for developing a new concept to solve this concern is therefore sufficiently justified.

For answering the above described parallelizability problem, several parallel concepts have been introduced, one good example being the so-called cellular neural network (CNN). It has been introduced by Leon Chua in 1988 in the seminal paper entitled: “Cellular Neural Networks: Theory” [30]. In 1993, a further article entitled: “The CNN Universal Machine: An Analogic Array Computer”, was published by Tamas Roska and Leon Chua, where the first analog CNN processor was presented [31]. Since then many different articles have been published to show the applicability of CNN processors for various usages.

In this article, we use a more generic concept which is called recurrent neural network (RNN), which is basically a more general neural network family to which CNN belongs. Essentially, they (i.e., RNN) are building elements of the so-called dynamic neural network (DNN) [32]. The parallel computing nature of RNN and the inherent fast processing speed while solving problems make it a very good basic brick of a novel concept for efficiently solving differential problems on multiple cores [33].

An RNN processor, similarly to its ancestors (artificial neural networks), is a set of cells, which do have mathematical relations (i.e., coupling) with their respective neighbors. The dynamic property of each cell is expressed by one differential equation. Furthermore, the dynamic property of the network can be customized for solving a given ODE (ordinary differential equation) by appropriate values of ODE’s parameter settings (or coefficients) expressed in the form of matrices called “templates”.

Playing with templates does provide the possibility to solve different kind of problems without changing the physical property and/or the architecture of the RNN machine or processor.

The inherent flexibility and the fast processing speed while solving problems with RNN does provide two advantages to solve problems when compared to traditional or competing computational methods or concepts [34,35,36]. First, we can solve different kind of problems by changing templates. Second, the problem solving can be significantly accelerated (see speed-up) without losing accuracy.

The above-mentioned good features of RNN (e.g., flexibility, speed-up (in presence of multiple cores), etc.) motivate the use of this promising paradigm for an accelerated solving of linear algebraic equations on either one-core or multi-core platforms. This is not a simple conversion as we do face completely different hardware computing frameworks with different parameters to be taken care of. All RNN parameters should be adjusted such that the resulting new dynamical system does converge to the target solution of the problem.

In this paper, we do need to formulate the central problem (i.e., realizing a “matrix inversion”) in a manner such that the final state of the RNN system is the solution of the target linear algebraic system of equations; see Equation (1).

(1) M (t) X=I

In more detail, we define the linear algebraic equations system as Equation (1). Hereby, M(t) is a n×n matrix of smooth time varying real numbers and X is a n×n matrix, I is identity matrix of size n×n. We would like to find X such that Equation (1) is satisfied. Our target is to find corresponding RNN templates, which are such that for any initial value problem (IVP), after a finite number of iterations, our dynamic system (see Equation (2)) converges to a solution $X^{*}$, which approximates X.

(2) $$\dot{X}=F\left(X\right)\;\forall X\in {\mathbb{R}}_{n\times n},\underset{t\to \infty }{\lim}MX-I=0$$

In Equation (2), $\dot{X}=F\left(X\right)$ is showing an ODE in a general form. The second part of Equation (2) is showing our problem that we wish to convert into the form of an ODE solving.

There exist several published works, in which one has tried to solve similar problems with dynamical systems like recurrent neural networks (RNN) [13,37,38,39,40,41,42,43] or artificial neural networks (ANN) [44,45,46,47], etc. Although most of those works are also related to RNN, our work and the novel concepts presented in this paper have more potential to reliably provide faster convergence towards the solution of Equation (1).

One of the main differences between our method and other related works, especially RNN concepts, is that the proposed model does need training like this required for most of RNN networks required. Our model is converging to the problem’s solution if and only if a correct selection/setting of internal dynamic parameters is done. Therefore, this functionality makes our model completely unique amongst other types of RNN models, which are usually used in machine learning [48].

For training, most of the published related works use common traditional methods like gradient descent [39] to create dynamical systems and do then customize them for an implementation on a target platform.

In this paper we do compare the performance of the own novel method developed with those already implemented traditional methods from the relevant literature. This does provide the basis for a fair judgement of both advantages and disadvantages of each method, old ones versus our new one.

The overall structure of this paper is as follows. Section 2 contains both background and a comprehensive overview of previous dynamic system models used for matrix inversion. Besides, related requirements with respect to the target platform for implementing the dynamic system (RNN, ANN …) are discussed. In Section 3, we do formulate our problem (i.e., inverting a time-varying matrix) with required restrictions related to RNN. The effectiveness of the developed model for solving the matrix inversion problem will be demonstrated in Section 4. The main proof and interesting result are the one showing the global (fast) convergence of the RNN-based dynamic system to the final solution of Equation (1).

In Section 5, the new method will be used to create a new dynamical system, i.e., an RNN model. The performance of this novel RNN will be compared to that of previous/original concepts.

In the last section (i.e., Section 7), some concluding remarks summarize the quintessence of the key achievements in this work.

## 2. Related Works of Dynamical Neural Networks

Solving Equation (1) using any dynamic method like the so-called dynamic neural network (DNN) requires creating a dynamic system with an attractor in that system (if and only if Equation (1) has a real solution), which is a fix point equal to the solution of Equation (1). Such systems can be implemented by different ways, but the most famous way of creating such dynamic systems is to use either the gradient descent method, the Zhang dynamics, or the Chen dynamics. All three named methods do essentially use the same concept, but both the second and the third named methods do use smaller step sizes and therefore more memory is required. This can improve/speed-up the convergence of the algorithms. We will be providing (in the next sections) a full explanation of the advantages of each method; afterwards a generic new method for solving Equation (1) will be provided.

### 2.1. The Gradient Method

This method involves a first-order optimization technique for finding the minimum point of a function. This technique takes steps in the direction of the negative gradient. It is based on the observation of where the multivariate function F(X) decreases the fastest if we choose the negative gradient of F(X) at point a: −∇F(a).

(3) b=a−γ∇F(a), ∀γ∈ℝ and γ>0

γ is the step size for updating decision neurons. For small γ, F(a)≥F(b); with this remark, we can extend the observations by Equation (4).

(4) $$X_{n+1}=X_{n}-\gamma \nabla F\left(X_{n}\right)$$

That means, by iterating Equation (4), F(X) will be converging to the minimum point of the function F.

The gradient descent (see Equation (5)) can also be described as the “Euler method” for solving ordinary differential equations.

(5) $$\dot{X}\left(t\right)=-\gamma \nabla F\left(X\left(t\right)\right)$$

Gradient descent can be used to solve linear equations. In that case, the problem is reformulated as the quadratic minimization of a function which is then solving it with gradient descent method.

(6) $$F\left(X\right)=\frac{1}{2}{\left|\left|MX-I\right|\right|}^{2}$$

Then, based on Equation (6), we have:

(7) $$\nabla F\left(X\right)=M^{T}\left(MX-I\right)$$

By substituting Equation (7) into Equation (5) the following dynamic model is obtained:

(8) $$\dot{X}\left(t\right)=-\gamma M^{T}MX+\gamma M^{T}I$$

The exact analytical solution of Equation (8) is expressed as the following:

(9) $$X\left(t\right)=\left(X\left(0\right)-M^{-1}\right)\;e^{-\gamma M^{T}M.t}+M^{-1}$$

Whereby the first derivative of X(t) denoted by $\dot{X}\left(t\right)$ will be:

(10) $$\dot{X}\left(t\right)=-\gamma \left(X\left(0\right)-M^{-1}\right)\;\left(M^{T}M+{\dot{M}}^{T}Mt\right)\;e^{-\gamma M^{T}M.t}$$

Equations (8) and (1) converge to the same solution described by the point $M^{-1}$.

$$\forall \gamma ,a_{i,j}\in \mathbb{R}\;and\;\gamma >0,\;then\;\gamma MM^{T}>0$$

This method is also called “gradient-based” dynamics. It can be designed by norm-based energy functions [45,49]. The advantage of this model is its easiness of implementation, but due to the effective factor of convergence which can be seen in Equation (9) it takes (a long) time to converge to the solution of the problem, and this convergence rate has an effect on noise sensitivity of the model as it makes the model more sensitive to the noises. Also, this model is not appropriate for real-time matrixes.

### 2.2. Zhang Dynamics 

There exists another method to create [13,50] a dynamic system converging to the required solution. In this method, the error function is defined in the following way:

(11) E(t)=M(t)X(t)−I

This means the error will be increasing proportionally to the amount of divergence of *X* from its true solution. Larger values of the difference will create larger values of the error.

Equation (11) is changing over time in a way such that the result will be the same as Equation (2). This requires that one moves against errors to come closer to the solution. Therefore, one can define the derivation of this function as following:

(12) $$\dot{E}\left(t\right)=-\gamma E\left(t\right)$$

The solution of Equation (12) can be expressed as Equation (13). This equation is showing how the error function is decreasing exponentially in a reverse direction to the errors. This will force the function to converge to the solution. Using this dynamic system is helping to create a new dynamic system for our problem.

(13) $$E\left(t\right)=C\;e^{-\gamma t}$$

By substitution of E(t) and $E^{\prime }\left(t\right)$ into Equation (12) we will obtain a new dynamic system [11].

(14) $$M\dot{X}+\dot{M}X=-\gamma \left(MX-I\right)$$

Whereby the solution of Equation (14) expressed as follows:

(15) $$X\left(t\right)=Ce^{-\gamma t}+M^{-1}$$

When we make a first derivation of Equation (15) we will have:

(16) $$\dot{X}\left(t\right)=-C\gamma e^{-\gamma t}$$

By combining Equations (15) and (16) we derive Equation (14). It is observed again that by increasing t to infinite our dynamic system will be converging to the solution of Equation (1). This model has a very good convergence rate and this will solve the problem of noise sensitivity. On the other hand, it is a model which is much more difficult to implement.

### 2.3. Chen Dynamics

This method is a combination of the two previously described methods. It assumes matrix M is not changing during time; therefore, the time-derivation of M is zero. If we multiply Equation (8) by M and then sum it with Equation (14) we will obtain a new dynamic system [39]:

(17) $$M\dot{X}\left(t\right)=-\gamma M^{T}M\;\left(MX\left(t\right)-I\right)\;M\dot{X}\left(t\right)=-\gamma \left(M^{T}M+I\right)\left(MX-I\right)$$

Solving Equation (17) lead to the following solution:

(18) $$X\left(t\right)=-C\;M^{-1}e^{-(M^{T}M+I)t}+M^{-1}$$

A derivation of Equation (18) leads to following dynamical system:

(19) $$\dot{X}\left(t\right)=C\;\left(M^{T}+M^{-1}\right)\;e^{-(M^{T}M+I)t}$$

By combining Equation (17) in Equation (18) leads to Equation (19).

If $M^{T}M>0$ we can state and confirm that this method has a better convergence rate than the first and second above mentioned ones [39]. As we see in Equation (19) t is multiplied with $\left(M^{T}M+I\right)$ in the exponent term, which produces a larger exponent value than the ones for the previous two methods. This model is better than the previous model. It has a very good convergence rate and its sensitivity to noise is very low. On the other hand, its implementation is difficult as it has more coefficient to calculate with respect to the other methods and it does not fit for a real-time matrix inversion (i.e., for inverting a time-varying matrix).

### 2.4. Summary of the Main Previous/Traditional Methods

By comparing the properties of the above presented methods, there is one big difference amongst them. Table 1 shows the major differences between those models by using 4 different criteria. The convergence rate refers to the convergence during time during the solving process. The “time-varying time matrix inversion” criteria refers to the ability of a given model to be usable for the case of the inversion of a time-varying matrix. The “implementation” criteria refer to how easy it is to implement a given model on RNN machines/processors. And the last criteria in Table 1 refers to how far a given model is sensitive to noise that is present in the time-varying matrix values. This last-mentioned criterion is very important because it does express the resilience of a given model to noise, which is always present in analog computing signals. Although noise is relatively low in digital systems, digital systems do however introduce a noise-equivalent signal distortion originating from computational rounding of numbers as they are digitally represented and processed with/in a fix-size digital arithmetic.

The Zhang model does have a fixed convergence rate over to time. But the gradient descent model does contain a coefficient which can be changed to influence (increase or decrease) the convergence rate. However, the Chen model is much better than the two previous ones as it does potentially provide a much better convergence rate.

The convergence rate has a direct impact on the noise sensitivity of a given model. Indeed, an increase of the convergence rate does decrease the noise sensitivity. Therefore, the Chen model has highest level of stability with respect to noise, although it is the more complex to implement.

Furthermore, amongst the 3 models listed in Table 1, only the Zhang model offers the capability of solving a time-varying matrix (i.e., a real-time matrix inversion).

## 3. Our Concept: The Novel RNN Method

According to Chen [39], his model is converging to the solution of Equation (1) under any initial value. One can however re-formulate the Chen model [39] as result of following goal function:

(20) $$\min\;\{Z={\left\|\left(X-A^{-1}\right)\right\|}^{2}+{\left\|(AX-I)\right\|}^{2}\}$$

We can add another positive statement to Equation (20); thus, we multiply the last term of Equation (20), i.e., the term (${\left(AX-I\right)}^{2})$, with the matrix A and we add it again to the function Z.

(21) $$\min\;\{Z={\left\|(X-A^{-1})\right\|}^{2}+{\left\|\left(AX-I\right)\right\|}^{2}+{\left\|\left(A^{T}AX-A^{T}\right)\right\|}^{2}\}$$

After adding that new term to the right side of Equation (20) and solving Z (according to Equation (21), one does find/obtain the following dynamical system:

(22) $$M\dot{X}\left(t\right)=-\gamma \left({\left(M^{T}M\right)}^{2}+M^{T}M+I\right)\left(MX-I\right)-\dot{M}X$$

The solution of this equation (see Equation (22)) can be expressed as follows:

(23) $$X\left(t\right)=-C\cdot M{\left(t\right)}^{-1}e^{-\gamma \int_{0}^{t}\left({\left(M^{T}M\right)}^{2}+M^{T}M+I\right)dz}+M{\left(t\right)}^{-1}$$

In Equation (23), when times goes to infinite the limit of X will converge to $M{\left(t\right)}^{-1}$ and it thereby provides the solution for Equation (1). *C* is a constant value (a matrix), which is added during/while solving Equation (22); see Table 2 for an illustration. Newly added terms ${\left(M^{T}M\right)}^{2}+M^{T}M$ produce a better convergence rate. The main reason of this convergence rate is the positive value of the integral and it provides additional factors when compared to the previous time-varying models. Therefore, by adding more coefficients to the right-hand side of Equation (22), we can obtain the following equation:

(24) $$M\dot{X}\left(t\right)=-\gamma \left(\overset{n}{\underset{i=0}{\sum }}{\left(M^{T}M\right)}^{i}\right)\left(MX-I\right)-\dot{M}X$$

Equation (24) is more general and can create the model of Equation (22), which is a specific configuration of it. For this, we just need to set the value of parameter n to 2.

**Theorem** **1.** For any given nonsingular matrix $M\in {\mathbb{R}}^{n\times n}$, the state matrix $X\left(t\right)\in {\mathbb{R}}^{n\times n}$, while starting from any (initial value problem) IVP $X\left(0\right)\in \;{\mathbb{R}}^{n\times n}$, Equation (24) will achieve global convergence to $X^{*}\left(t\right)=M^{-1}\left(t\right)$.

**Proof** **of** **Theorem 1.** Let define $E\left(t\right)=X\left(t\right)-X^{*}\left(t\right)$ be the error value during the process for finding the solution. If this equation is multiplied by M, it does lead to $M\left(t\right)E\left(t\right)=M\left(t\right)X\left(t\right)-M\left(t\right)X^{*}\left(t\right)$ or M(t)E(t)=M(t)X(t)−I. Thus, a derivation of the error function will lead to $M\dot{E}\left(t\right)+\dot{M}E\left(t\right)=M\dot{X}\left(t\right)-\dot{M}X\left(t\right).$ By replacing this in Equation (24) we obtain the following expression:

(25) $$M\dot{E}\left(t\right)=-\gamma \left(\sum_{i=0}^{n}{\left(M^{T}M\right)}^{i}\right)M\;E\left(t\right)-\dot{M}\;E\left(t\right)$$

Let us define the Lyapunov function ϵ(t)=ME(t), which is always a positive function. The derivative of this function can be obtained as follows:

(26) $$\dot{\epsilon }\left(t\right)=E{\left(t\right)}^{T}M^{T}M\cdot \frac{dE\left(t\right)}{dt}+E{\left(t\right)}^{T}M^{T}\frac{dM\left(t\right)}{dt}E\left(t\right)$$

By replacing Equation (25) into Equation (26) it leads to the following:

(27) $$\dot{\epsilon }\left(t\right)=-\gamma E{\left(t\right)}^{T}(\overset{n}{\underset{i=0}{\sum }}{\left(M^{T}M\right)}^{i+1})E\left(t\right)$$

Hence:

(28) $$\dot{\epsilon }\left(t\right)=-\gamma E{\left(t\right)}^{T}M^{T}(\overset{n}{\underset{i=0}{\sum }}{\left(M^{T}M\right)}^{i})ME\left(t\right)$$

One can replace middle term $\overset{n}{\underset{i=0}{\sum }}{\left(M^{T}M\right)}^{i}$ with large enough value of µ therefore:

(29) ≤−γµ ‖ME(t)‖≤0

Thus, it appears that $\dot{\epsilon }\left(t\right)$ is always negative; furthers, $\dot{\epsilon }\left(t\right)=0$ if and only if $X\left(t\right)=X{\left(t\right)}^{*}$ is satisfied. Therefore, our differential equation globally converges towards a point (matrix), which is the equilibrium point for this function. □

Equation (30) is the result of an analytical solving of Equation (24). Increasing t in this equation will lead to the solution of the algebraic equation (i.e., of Equation (1)).

(30) $$X\left(t\right)=-C\cdot M^{-1}e^{-\gamma \sum_{i=0}^{n}\int_{0}^{t}{\left(M^{T}M\right)}^{i}dz}+M^{-1}$$

In this equation, C is a constant value (matrix) and it is added during solving differential equation. Obviously, this equation has a much better rate of convergence when compared to the previous implementations and, like previous solutions/concepts, the minimum value of the eigenvectors of $M^{T}M$ should be positive.

Also, according to the Chen model, if Equation (24) is extended by introducing a monotonically increasing function F where F(0)=0, here again our system will be converged to solution of Equation (1). Thus, by introducing a function F in the Equation (24) the following new equation will be obtained, see Equation (31):

(31) $$M\dot{X}\left(t\right)=-\gamma \left(\overset{n}{\underset{i=0}{\sum }}{\left(M^{T}M\right)}^{i}\right)F\left(MX-I\right)-\dot{M}X$$

**Theorem** **2.** For any given nonsingular matrix $M\in {\mathbb{R}}^{n\times n}$, the state matrix $X\left(t\right)\in {\mathbb{R}}^{n\times n}$, while starting from any IVP (initial value problem) $X\left(0\right)\in {\mathbb{R}}^{n\times n}$ and with a monotonically increasing function F where F(0)=0, Equation (31) will achieve global convergence to $X^{*}\left(t\right)=M^{-1}\left(t\right)$.

**Proof** **of** **Theorem 2.** Let define $E\left(t\right)=X\left(t\right)-X^{*}\left(t\right)$ for the error value during the process for finding the solution. If this equation is multiplied by M, it does lead to $M\left(t\right)E\left(t\right)=M\left(t\right)X\left(t\right)-M\left(t\right)X^{*}\left(t\right)$ or M(t)E(t)=M(t)X(t)−I. Thus, a derivation of the error function will lead to $M\dot{E}\left(t\right)+\dot{M}E\left(t\right)=M\dot{X}\left(t\right)-\dot{M}X\left(t\right).$ By replacing this in Equation (31), we obtain the following expression:

(32) $$M\dot{E}\left(t\right)=-\gamma \left(\overset{n}{\underset{i=0}{\sum }}{\left(M^{T}M\right)}^{i}\right)\;F\left(ME\left(t\right)\right)-\dot{M}\;E\left(t\right)$$

Let’s define the Lyapunov function ϵ(t)=ME(t), which is always a positive function. The derivative of this function can be obtained as follows:

(33) $$\dot{\epsilon }\left(t\right)=E{\left(t\right)}^{T}M^{T}M\cdot \frac{dE\left(t\right)}{dt}+E{\left(t\right)}^{T}M^{T}\frac{dM\left(t\right)}{dt}E\left(t\right)$$

By replacing Equation (33) into Equation (32) it does lead to:

(34) $$\dot{\epsilon }\left(t\right)=-\gamma E{\left(t\right)}^{T}M^{T}(\overset{n}{\underset{i=0}{\sum }}{\left(M^{T}M\right)}^{i})F\left(ME\left(t\right)\right)$$

Hence:

(35) $$\dot{\epsilon }\left(t\right)=-\gamma E{\left(t\right)}^{T}M^{T}(\overset{n}{\underset{i=0}{\sum }}{\left(M^{T}M\right)}^{i})F\left(ME\left(t\right)\right)$$

One can replace middle term $\sum_{i=0}^{n}{\left(M^{T}M\right)}^{i}$ with large enough value of µ therefore:

(36) $$\leq -\gamma µ\;E{\left(t\right)}^{T}M^{T}F\left(ME\left(t\right)\right)\leq 0$$

In the last equation, $E{\left(t\right)}^{T}M^{T}F\left(ME\left(t\right)\right)$ is always positive because if ME(t) becomes negative, F(ME(t)) also becomes negative and vise-versa.

Thus, it appears that $\dot{\epsilon }\left(t\right)$ is always negative; and $\dot{\epsilon }\left(t\right)=0$ if and only if $X\left(t\right)=X{\left(t\right)}^{*}$ is satisfied. Therefore, our differential Equation (31) or (32) globally converges towards a point (matrix), which is the equilibrium point for this function. □

By choosing different forms of the function F, one can create various dynamical properties to be expressed by this model.

**Figure 1:**
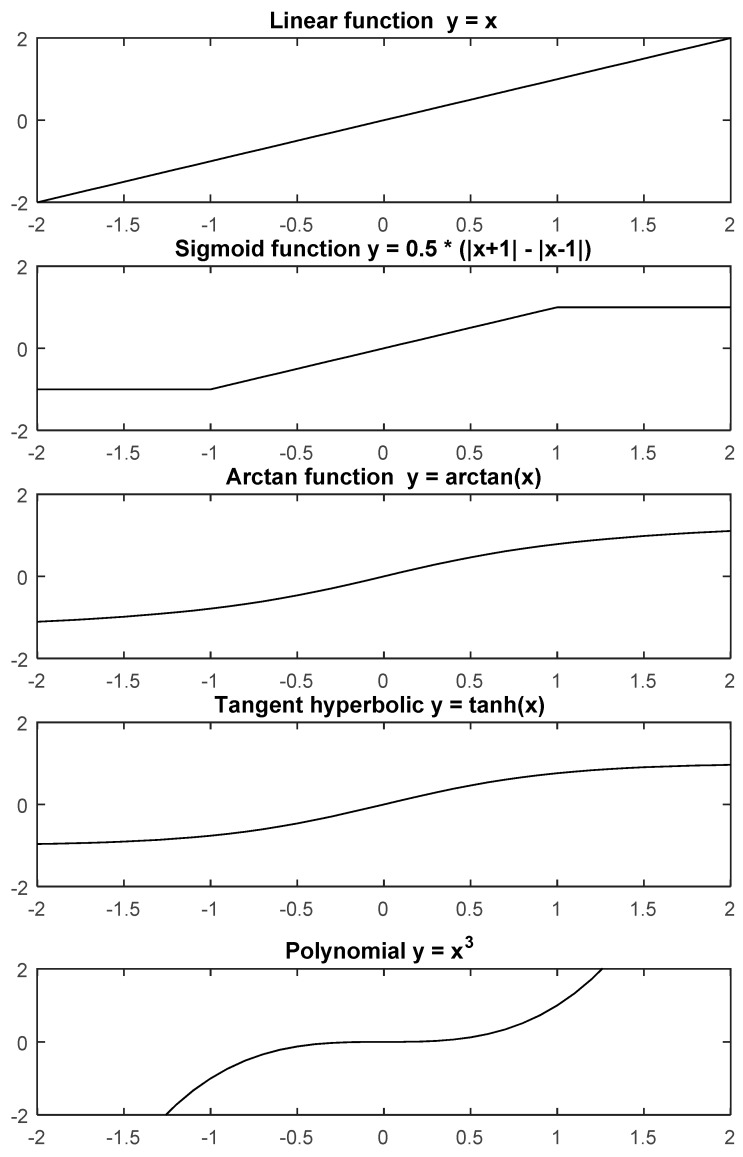
Illustrative examples of monotonic functions which can be used for solving the inversion of a time-varying matrix through Equation (31).

Examples of functions for F are: sigmoid, linear, square, cubic, arcos, etc. All these functions are suitable to be used in Equation (31) as all of them are increasing monotonic functions and they do all satisfy the F(0)=0 condition (see Figure 1).

## 4. Model Implementation in SIMULINK

**Figure 2:**
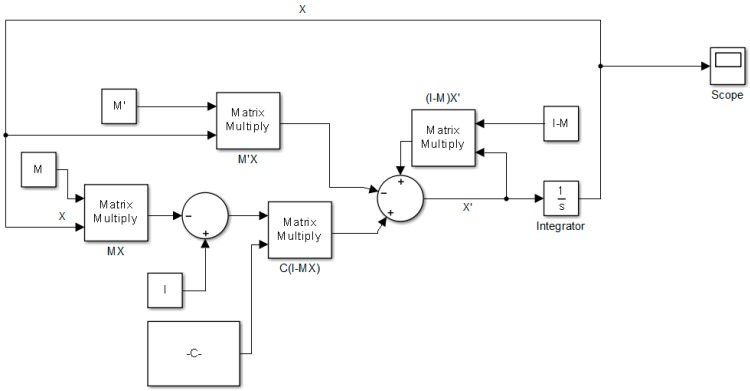
The RNN block diagram corresponding to Equation (24). Note that the matrix to be inverted is M.

Equation (24) or Equation (31) can be implemented directly into SIMULINK (see Figure 2). This dynamic model has the components shown in Table 2.

**Figure 3:**
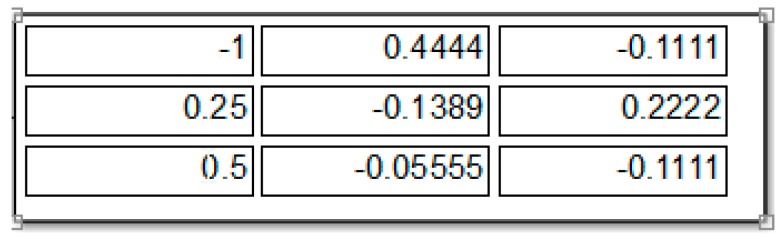
Result of model simulation (just for illustration) for the matrix M indicated in Table 2 (see first row of Table 2), SIMULINK output.

If M is not a time-varying matrix we can simply put zero values in the M’ matrix, otherwise M’ model should be a corresponding derivative of matrix M. Executing the model will result in the following output in SIMULINK, see Figure 2, Figure 3, and Table 2, which is the solution of Equation (1). Therefore, it works well as we expected and gives the solution of Equation (24).

## 5. Illustrative Examples

In this section we consider some numerical examples to demonstrate the performance of our RNN implementation for solving a system of linear algebraic equations.

### 5.1. Illustrative Example 1

Let us define M and V as follows: Here, instead of using the identity matrix in Equation (1) we use a vector V. This does result in a linear system of equations.

(37) $$M=\left[\begin{array}{ccc} -3.0755 & 0.5004 & 3.8135\\ 0.0739 & -2.2382 & -4.7532\\ -4.7576 & 1.9624 & -1.5879 \end{array}\right],\;V=\left[\begin{array}{c} 7.920\\ 7.983\\ 9.633 \end{array}\right]$$

Thus, one can find X as following:

(38) $$X=M^{-1}V=\left[\begin{array}{c} -3.330\\ -3.305\\ -0.175 \end{array}\right]$$

This above given value of X is the target value (ground truth). In the following, we shall see how far how fast the models developed (Equation (24) and/or Equation (31)) do reach well this target value.

M is not singular and therefore can be inverted. Thus, our system of equations has one unique solution. C, as explained in the previous section, is a weight values-matrix which is calculated using the following formula:

(39) $$C=\overset{n}{\underset{i=0}{\sum }}{\left(M^{T}M\right)}^{i}$$

Increasing the value of n will increase the convergence rate of Equation (24) or Equation (31) or (29). Here, we choose the value n = 3. The corresponding RNN parameters are defined as follows (see Figure 3):

(40) $$C=M^{T}MM^{T}MM^{T}M+M^{T}MM^{T}M+M^{T}M+I\;C=10,000\times \left[\begin{array}{ccc} 4.6283 & -2.2035 & -2.7439\\ -2.2035 & 1.3283 & 2.5763\\ -2.7439 & 2.5763 & 7.5178 \end{array}\right]\;X_{0}=\left[\begin{array}{c} 0\\ 0\\ 0 \end{array}\right]$$

These parameters are implemented in the previously described model in Simulink (see Figure 3) for numerical simulations and F (for simplicity) is taken as a linear function (Figure 1).

**Figure 4:**
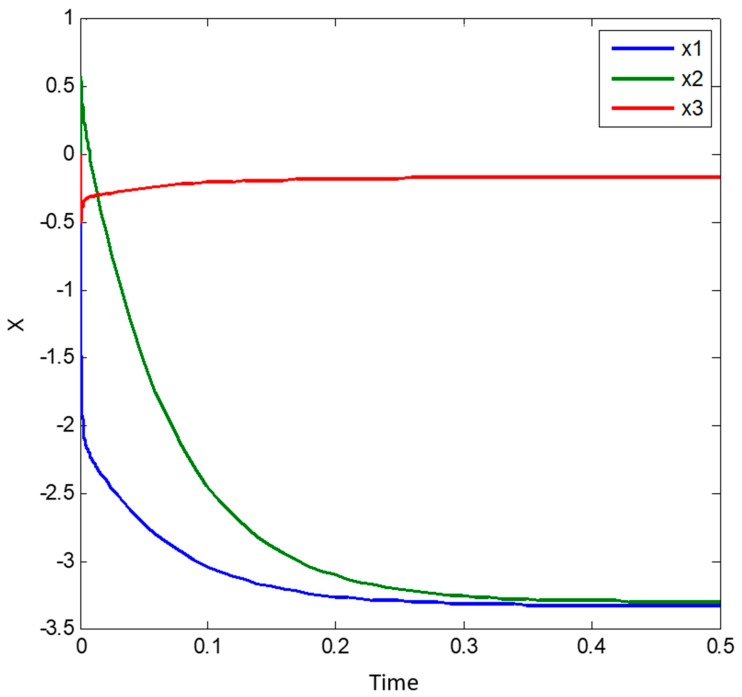
Result of our model simulation for solving the illustrative example with respect to solving an algebraic system of linear equations. Please note in this case we have taken n = 3 in Equation (24) or Equation (31).

Figure 4 is showing the system simulation results during the time interval t=[0, 0.5]. The output (value of state vector) at the end of this time interval is the following:

(41) $$X\left(0.5\right)=\left[\begin{array}{c} -3.329\\ -3.304\\ -0.1749 \end{array}\right]$$

This above obtained result shows that the output of our system model is very close to the analytical solution and the difference is small and approximately 0.001, which can furthermore be corrected but adjusting the step size.

### 5.2. Illustrative Example 2

Now we do the same experience for calculating the inverse of a time-varying matrix *M*. The matrix *M* is defined as follows:

(42) $$M=\left[\begin{array}{cc} Sin\left(t\right) & -Cos\left(t\right)\\ Cos\left(t\right) & Sin\left(t\right) \end{array}\right]$$

the matrix *M* is always invertible and it does satisfy the conditions of Equation (1). Therefore, we can use it for testing our dynamic system model.

(43) $$M^{T}=M^{-1}=\left[\begin{array}{cc} Sin\left(t\right) & Cos\left(t\right)\\ -Cos\left(t\right) & Sin\left(t\right) \end{array}\right]$$

Also, we define F as a linear function (Figure 1).

As we have explained previously (see Table 2), we calculate the weight *C* again for n = 3 as the following:

(44) $$C=M^{T}MM^{T}MM^{T}M+M^{T}MM^{T}M+M^{T}M+I$$

**Figure 5:**
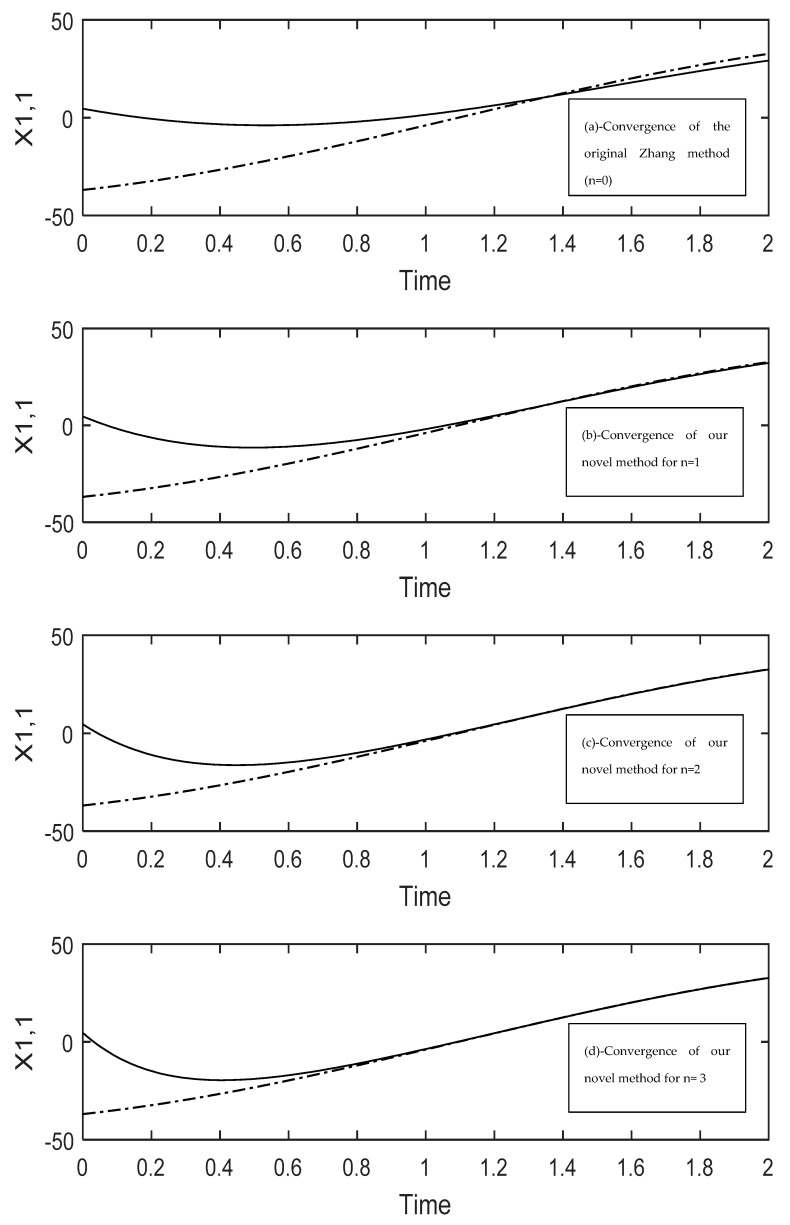
Showing the difference in convergence speed between the original Zhang method and our new developed method (see Equation (24)). The solid lines are the one obtained from the model solving and the dashed lines are the target values. (**a**) convergence of the original Zhang model; (**b**) until (**d**): convergence profiles of our novel method for different values of n. All graphs (a) until (d) are plotted for the first element of the time-varying matrix M (i.e., first row, first column).

Now all parameters are introduced in the Simulink model to simulate our system model (see Equation (24)). The simulation is done for t=[0, 2.0]. Figure 5 does contain benchmarking results for the first element of the time-varying matrix (i.e., the matrix element in first row, first column). Here (see Figure 5) the speed of convergence is compared to that of the original Zhang model. Thereby, for our model we consider different values of the parameter n. One can clearly see that our novel model strongly outperforms the original Zhang model. Table 3 shows the difference very clearly: Here, it is clear that by choosing n = 3 our model will converge to the problem solution 4 times faster than the ZNN model; notice that the ZNN model corresponds to n = 0 in Table 3. Amount of memory for saving this model is very small as we need to save main templates parameter of dynamic model in Equation (24) or Equation (31). Therefore, as see in Table 3, the memory usage is very small and it can be implemented on small computers.

### 5.3. Illustrative Example 3

In the last illustrative experiment, we try to test and analyze the effect of changing the function F in Equation (31) on the convergence rate of our model.

**Figure 6:**
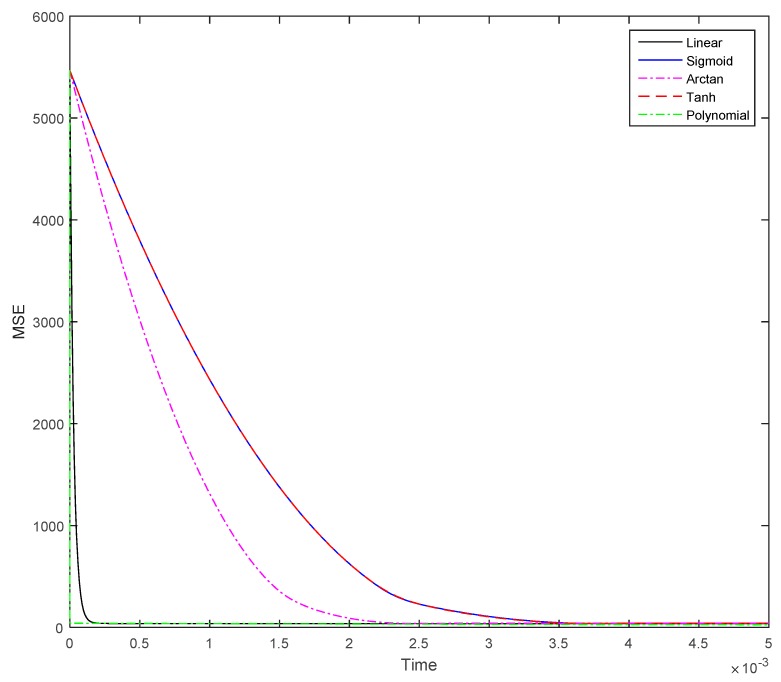
Showing differences in convergence speed while considering different function types for F in Equation (31). Please note that n is 3 for the polynomial function F.

For this test, we take the same parameter setting as in the first experience (see illustrative example 1), but we change the function F and calculate coefficients for n=3. The selected functions for this test are the following ones: linear function (Y=X), sigmoid function ($Y=\frac{1}{2}(\left|X+1\right|-\left|X-1\right|$), arctangent function (Y=arctan(X)), tangent hyperbolic function (Y=tanh(X)), and polynomial function ($Y=X^{3})$. All other parameters are fixed such to show the correct properties of the functions. As we can see in the results of this simulation experiment (see Figure 6) the polynomial function is showing the very best convergence amongst all considered functions. Therefore, it is evident that selecting the appropriate function can help our model to converge faster to the solution of the matrix inversion problem. It is also evident that higher values of n will perform much better.

Table 4 is showing our simulation result until t = 0.05 for this illustrative example 3. It does clearly show the effect of using different functions on the system/model convergence. Both polynomial and linear functions are the best functions for this task, while both sigmoid and tanh functions are the worst functions to be used in this context for matrix inversion.

The superiority with respect to convergence speed of the polynomial function F is very evident.

## 6. Comparison of Our Novel Method with Previous Studies

**Figure 7:**
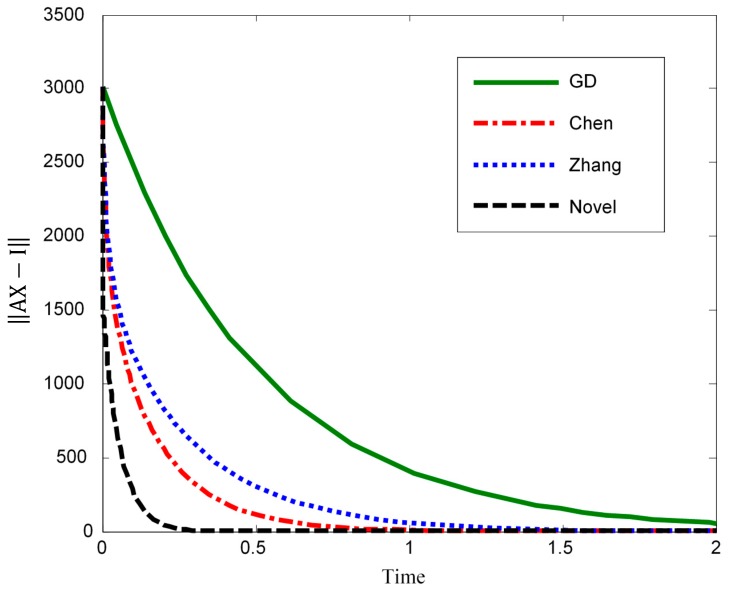
Comparison of different DNN models with respect to convergence speed under the same conditions—benchmarking. In this figure, GD refers to the “gradient descent” method. Our novel method (see novel in the figure; Equation (24)) is calculated for n = 3. The fast convergence of our method to the exact solution is clearly demonstrated. It is also clear that this convergence would be much faster if higher values of n are taken (n > 3).

In this section we compare our novel model with the following related methods which are well-known from the relevant literature: gradient descent method, Zhang dynamics, and Chen dynamics. Hereby, 1000 different static random matrices are generated for use in the experiment. Finally, we then sum up the results obtained as shown in Figure 7. Figure 7 does display how the error converges towards zero when the different models are executed over time; the intention is to hereby illustrate the convergence speed of all considered models. Hereby, the error function is calculated by using the formula ‖MX−I‖.

By comparing the different methods with our method (Figure 7), the fastest convergence of our method to the exact solution is observed. Specifically, our method is at least 3 times faster than the Chen method while implemented on CPU (the results of Figure 7 were obtained on CPU). An implementation on a multiple-core platform should display a much higher relative and comparative speed-up performance of our model. Obviously, by using more coefficients (i.e., higher values of n) in Equation (24) we can reach a much better convergence rate.

## 7. Conclusions

A novel method for solving the very important “matrix inversion” problem has been developed and validated in this paper. Consequently, it can also solve linear algebraic systems of equations. The concept developed is essentially an RNN-based analog computing machine.

We have compared this novel method with other dynamical systems methods from the relevant literature. It has been demonstrated that our novel method does theoretically have an exponential convergence rate towards the exact solution of the problem for any IVP (initial value problem). Also, the convergence rate of this method is much higher than those of other related competing concepts.

It is further possible to customize this novel model in order to enable its implementation on a cellular neural network processor machine. This has previously been done in our previous works.

For validation we have extensively compared our method with other relevant competing methods like gradient descent, Zhang, and Chen methods in one large simulation experiment. Hereby, we have considered the following scenarios: 1000 random matrixes of M are generated and applied in the different dynamical system models for matrix inversion. For each time-point, errors reached are summed-up using the formula ‖MX−I‖.

It has been clear that while using our novel method we do visibly reach a significant speed-up even on one single CPU, this compared to the other methods. A use of more coefficients (i.e., higher values for n in model Equations (24) and (31)) will surely result in an even much higher convergence rate. On the other hand, however, if we use more coefficients, we would need more preparation time to create the templates and consume more computing resources for running the (our novel) RNN processor dynamical model. Therefore, it is recommended to reach a balance/tradeoff between convergence rate and number of required coefficients (i.e., value of n).

To finish, the last experiments have demonstrated that using the polynomial function for F in Equation (31) does lead to a clearly much higher convergence rate when compared to the other types of function. Further, the higher the polynomial order, the best.

## Figures and Tables

**Table 1:** Comparison of different types of DNN (dynamic neural network) concepts (the traditional ones) for matrix inversion.

Criteria/Method	Gradient NN	Zhang NN	Chen
Convergence rate	$e^{-\gamma M^{T}Mt}$	$e^{-t}$	$e^{-\gamma (M^{T}M+I)t}$
Time-varying matrix inversion	not available	Available	not available
Implementation	Easy	Hard	very hard/difficult
Noise sensitivity	High	Low	very low

**Table 2:** Components of SIMULINK model to implement Equation (24).

Component	Description
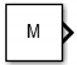	$$\left[\begin{array}{ccc} 1 & 2 & 3\\ 5 & 6 & 7\\ 2 & 6 & 1 \end{array}\right]$$
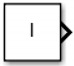	$$\left[\begin{array}{ccc} 1 & 0 & 0\\ 0 & 1 & 0\\ 0 & 0 & 1 \end{array}\right]$$
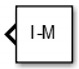	I-M
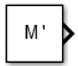	Time-derivative of M; if M is constant, just put zero values in the matrix M’: $$\left[\begin{array}{ccc} 0 & 0 & 0\\ 0 & 0 & 0\\ 0 & 0 & 0 \end{array}\right]$$
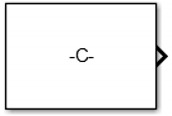	C is a weight value matrix equal to $\sum_{i=0}^{n}{\left(M^{T}M\right)}^{i}\left(see\;Equations\;\left(24\right)\;and\;\left(31\right)\right)$ For the setting n= 2, we do have the following value for C: $$M^{T}MM^{T}M+M^{T}M+I$$ Note: If the matrix M is time-varying, C will no more be constant and will also be time-varying.

**Table 3:** Comparison of performance of time varying matrix inversion with change of parameter n of Equation (24) or Equation (31). Please note n = 0 is ZNN model.

Criteria/n	0	1	2	3
**Convergence Time to MSE 0.01**	8.4	3.8	2.8	1.9
**MSE in t = 2.0**	3.4	0.4	0.06	0.008
**Approximated estimation of memory usage in Bytes**	96	128	128	128

**Table 4:** Performance comparison of different function types used in Equation (31), whereby all are monotonic increasing functions. The polynomial function shows the better convergence compared to the other functions.

Criteria/ Function used in Equation (31) with n = 3	Linear	Sigmoid	Arctan	Tanh	$X^{3}$
MSE at t = 0.05	0.075	7.90	4.58	7.83	0.062

## References

[1] Lumpkin B. (1997). Algebra Activities from Many Cultures.

[2] Song W., Wang Y. (2015). Locating Multiple Optimal Solutions of Nonlinear Equation Systems Based on Multiobjective Optimization. IEEE Trans. Evol. Comput..

[3] Wang Y., Leib H. (2013). Sphere Decoding for MIMO Systems with Newton Iterative Matrix Inversion. IEEE Commun..

[4] Gu B., Sheng V. (2013). Feasibility and Finite Convergence Analysis for Accurate On-Line ν-Support Vector Machine. IEEE Trans. Neural Netw. Learn. Syst..

[5] Eilert J., Wu D., Liu D. Efficient Complex Matrix Inversion for MIMO Software Defined Radio. Proceedings of the IEEE International Symposium on Circuits and Systems.

[6] Wu M., Yin B., Vosoughi A., Studer C., Cavallaro J.R., Dick C. Approximate matrix inversion for high-throughput data detection in the large-scale MIMO uplink. Proceedings of the ISCAS2013.

[7] Ma L., Dickson K., McAllister J., McCanny J. (2011). QR Decomposition-Based Matrix Inversion for High Performance Embedded MIMO Receivers. IEEE Trans. Signal Process..

[8] Arias-García J., Jacobi R.P., Llanos C.H., Ayala-Rincón M. A suitable FPGA implementation of floating-point matrix inversion based on Gauss-Jordan elimination. Proceedings of the VII Southern Conference on Programmable Logic (SPL).

[9] Irturk A., Benson B., Mirzaei S., Kastner R. An FPGA Design Space Exploration Tool for Matrix Inversion Architectures. Proceedings of the Symposium on Application Specific Processors.

[10] Benesty J. (2001). Adaptive eigenvalue decomposition algorithm for passive acoustic source localization. J. Acoust. Soc. Am..

[11] Warp R.J., Godfrey D., Dobbins J.T. (2000). Applications of matrix inversion tomosynthesis. Med. Imaging.

[12] Godfrey D., McAdams H.P., Dobbins J.T. (2013). The effect of averaging adjacent planes for artifact reduction in matrix inversion tomosynthesis. Med Phys..

[13] Zhang Y., Ge S. (2005). Design and analysis of a general recurrent neural network model for time-varying matrix inversion. IEEE Trans. Neural Netw..

[14] Guo D., Zhang Y. (2012). Zhang neural network, Getz–Marsden dynamic system, and discrete-time algorithms for time-varying matrix inversion with application to robots’ kinematic control. Neurocomputing.

[15] Guo D., Li K., Yan L., Nie Z., Jin F. The application of Li-function activated RNN to acceleration-level robots’ kinematic control via time-varying matrix inversion. Proceedings of the Chinese Control and Decision Conference (CCDC).

[16] Amato F., Moscato V., Picariello A., Sperlí G. Recommendation in Social Media Networks. Proceedings of the IEEE Third International Conference on Multimedia Big Data (BigMM).

[17] Amato F., Castiglione A., Moscato V., Picariello A., Sperli G. (2018). Multimedia summarization using social media content. Multimed. Tools Appl..

[18] Hopf B., Dutz F., Bosselmann T., Willsch M., Koch A., Roths J. (2018). Iterative matrix algorithm for high precision temperature and force decoupling in multi-parameter FBG sensing. Opt. Express.

[19] Cheng Y., Tsai P., Huang M. (2016). Matrix-Inversion-Free Compressed Sensing with Variable Orthogonal Multi-Matching Pursuit Based on Prior Information for ECG Signals. IEEE Trans. Biomed. Circuits Syst..

[20] Ji S., Dunson D., Carin L. (2009). Multitask Compressive Sensing. IEEE Trans. Signal Process..

[21] Bicchi A., Canepa G. (1994). Optimal design of multivariate sensors. Meas. Sci. Technol..

[22] Mach D., Koshak W.J. (2007). General matrix inversion technique for the calibration of electric field sensor arrays on aircraft platforms. J. Atmos. Ocean. Technol..

[23] Liu S., Chepuri S.P., Fardad M., Maşazade E., Leus G., Varshney P.K. (2016). Sensor Selection for Estimation with Correlated Measurement Noise. IEEE Trans. Signal Process..

[24] Zhang Y., Chen K., Tan H. (2009). Performance Analysis of Gradient Neural Network Exploited for Online Time-Varying Matrix Inversion. IEEE Trans. Autom. Control.

[25] Zhang Y., Ma W., Cai B. (2009). From Zhang Neural Network to Newton Iteration for Matrix Inversion. IEEE Trans. Circuits Syst..

[26] Kandasamy W., Smarandache F. (2012). Exploring the Extension of Natural Operations on Intervals, Matrices and Complex Numbers.

[27] Chen Y., Yi C., Qiao D. (2013). Improved neural solution for the Lyapunov matrix equation based on gradient search. Inf. Process. Lett..

[28] Yi C., Chen Y., Lu Z. (2011). Improved gradient-based neural networks for online solution of Lyapunov matrix equation. Inf. Process. Lett..

[29] Wilkinson J. (1961). Error Analysis of Direct Methods of Matrix Inversion. JACM.

[30] Chua L., Yang L. (1988). Cellular Neural Networks: Theory. IEEE Trans. Circuits Syst..

[31] Roska T., Chua L. (1993). The CNN universal machine: An analogic array computer. IEEE Trans. Circuits Syst..

[32] Endisch C., Stolze P., Hackl C.M., Schröder D. (2009). Comments on Backpropagation Algorithms for a Broad Class of Dynamic Networks. IEEE Trans. Neural Netw..

[33] Potluri S., Fasih A., Kishore L., Machot F.A., Kyamakya K. CNN Based High Performance Computing for Real Time Image Processing on GPU. Proceedings of the Nonlinear Dynamics and Synchronization (INDS) & 16th Int’l Symposium on Theoretical Electrical Engineering (ISTET).

[34] Mainzer K. CNN and the Evoulution of Complex Information Systems in Nature and Technology. Proceedings of the 7th IEEE Cellular Neural Networks and Their Applications.

[35] Chedjou J., Kyamakya K., Khan U., Latif M. Potential Contribution of CNN-based Solving of Stiff ODEs & PDEs to Enabling Real-Time Computational Engineering. Proceedings of the 2010 12th International Workshop on Cellular Nanoscale Networks and their Applications (CNNA 2010).

[36] Chedjou J., Kyamakya K. (2015). A Universal Concept Based on Cellular Neural Networks for Ultrafast and Flexible Solving of Differential Equations. IEEE Trans. Neural Netw. Learn. Syst..

[37] Stanimirovic P. (2015). Recurrent Neural Network for Computing the Drazin Inverse. IEEE Trans. Neural Netw. Learn. Syst..

[38] Wang J. (1997). Recurrent Neural Networks for Computing Pseudoinverses of Rank-Deficient Matrices. Siam J. Sci. Comput..

[39] Chen K. (2013). Recurrent Implicit Dynamics for Online Matrix Inversion. Appl. Math. Comput..

[40] Feng F., Zhang Q., Liu H. A Recurrent Neural Network for Extreme Eigenvalue Problem. Proceedings of the ICIC 2005.

[41] Tang Y., Li J. (2010). Another neural network based approach for commuting eigenvalues and eigenvectors of real skew-symmetric matrices. Comput. Math. Appl..

[42] Xu L., King I. (2001). A PCA approach for fast retrieval of structural patterns in attributed graphs. IEEE Trans. Syst. Man Cybern..

[43] Liu Y., You Z., Cao L. (2007). A recurrent neural network computing the largest imaginary or real part of eigenvalues of real matrices. Comput. Math. Appl..

[44] Bouzerdorm A., Pattison T. (1993). Neural Network for Quadratic Optimization with Bound Constraints. IEEE Trans. Neural Netw..

[45] Zhang Y. Towards piecewise-linear primal neural networks for optimization and redundant robotics. Proceedings of the IEEE International Conference on Networking, Sensing and Control.

[46] Hopfield J.J., Tank D. (1984). Neural Computation of Decisions in Optimization Problems. Cybernetics.

[47] Kenndey M.P. (1988). Neural Networks for Nonlinear Programming. IEEE Trans Circuits Syst..

[48] He Z., Gao S., Xiao L., Liu D., He H., Barber D. Wider and deeper, cheaper and faster: Tensorized LSTMs for sequence learning. Proceedings of the Conference on Neural Information Processing Systems 2017.

[49] Jang J.-S., Lee S.-Y., Shin S.-Y. An Optimization Network for Matrix Inversion. Proceedings of the 1987 IEEE Conference on Neural Information Processing Systems.

[50] Zhang Y., Li Z., Li K. (2011). Complex-valued Zhang neural network for online complex-valued time-varying matrix inversion. Appl. Math. Comput..

